# SARS-CoV-2 Infection in Spondyloarthritis Patients Treated With Biotechnological Drugs: A Study on Serology

**DOI:** 10.3389/fimmu.2021.682850

**Published:** 2021-06-11

**Authors:** Augusta Ortolan, Mariagrazia Lorenzin, Chiara Cosma, Giacomo Cozzi, Andrea Padoan, Andrea Doria, Mario Plebani, Roberta Ramonda

**Affiliations:** ^1^ Rheumatology Unit, Department of Medicine DIMED, University of Padova, Padova, Italy; ^2^ Department of Laboratory Medicine, University-Hospital of Padova, Padova, Italy; ^3^ Department of Medicine DIMED, University of Padova, Padova, Italy

**Keywords:** spondyloarthritis, SARS-CoV-2, COVID-19, serology, immunosuppression

## Abstract

**Objective:**

Serology could help to define the real extent of SARS-CoV-2 diffusion, especially in individuals considered at higher risk of COVID-19, such as spondyloarthritis (SpA) patients undergoing immunosuppressant. Our aim was to detect, by serology, previous SARS-CoV-2 contact in SpA, compared to health care workers (HCW), and healthy controls.

**Methods:**

Sera from consecutive patients affected by SpA undergoing cytokine-targeted therapy, HCW and healthy controls from 2015 were analysed through chemiluminescent analytical system for the presence of IgG and IgM anti-SARS-CoV-2. Positive patients (IgM or IgG, or both) additionally underwent real-time Polymerase-Chain-Reaction (RT-PCR) to test for active infection. Serology was repeated at 3-months in SpA. Data across 3 groups were compared by Kruskal Wallis/Chi-square, and between 2 groups by Wilcoxon rank test/Chi-Square. P ≤ 0.05 were considered significant.

**Results:**

200 SpA, 95 HCW and 101 controls were recruited. Positive serology was found in 25(12.5%) SpA, 8(8.4%) HCW, 0(0%) controls (p=0.001). SpA patients with positive serology more frequently reported COVID-19-like symptoms than those with negative serology (20% *vs.* 4%, p=0.009) and 2 had COVID-19 as confirmed by RT-PCR, non severe. No HCW reported symptoms or had positive RT-PCR. In SpA patients, at 3 months, mean IgM titres decreased from 2.76 ± 2.93 to 2.38 ± 2.95 (p=0.001), while IgG titres from 0.89 ± 3.25 to 0.31 ± 0.87 (p=ns).

**Conclusions:**

Serology revealed that exposure to SARS-CoV-2 in SpA patients and HCW was higher than expected based on reported symptoms. In SpA, anti-cytokine therapy could act as a protective factor for a severe disease course. However, a seroconversion was not observed at 3-months.

## Introduction

Since the pandemic outbreak of Severe Acute Respiratory Syndrome virus (SARS-CoV-2) in late 2019, there have been growing concerns about the susceptibility of patients undergoing immunosuppressive treatments (1). In theory, immunosuppressed patients could be at a higher risk of SARS-CoV-2 infection (COVID-19). However, some data suggest that these patients, in particular those affected by chronic arthritis undergoing cytokine-targeted therapies, might present a milder disease course (2). This could happen because pro-inflammatory cytokines such as interleukins 1β (IL-1β), IL-6, and Tumor Necrosis Factor alpha (TNFα) appear to play a detrimental role in COVID-19 (3). Notably, serum levels of certain cytokines allow to discriminate between mild, moderate, and severe cases (4). Thus, cytokine-targeted therapies may attenuate cytokine response resulting in a drop in intensive care unit admissions and mortality (1, 2).

In order to improve detection COVID-19 in non-severe patients, screening through serological testing could be applied. Unlike Real-Time Polymerase Chain Reaction (RT-PCR), the diagnostic gold standard for active SARS-CoV-2 infection, which requires active virus shedding, serological tests present the advantage of identifying individuals who have been previously infected (including mild/subclinical infections) and may help identify at-risk populations (5). In addition, some authors reported a positive correlation between anti-SARS-CoV-2 antibody titre and COVID-19 severity (6).

Based on these considerations, we set out to perform a pilot serological screening on consecutive spondyloarthritis (SpA) patients treated with cytokine-targeted biotechnological agents. We compared data from our patients with control sera from health care workers (HCW) and sera collected in 2015.

## Methods

### Patients

Consecutive patients with a diagnosis of axial or peripheral spondyloarthritis by the rheumatologist, classified as axSpA or peripheral SpA according to the ASAS classification criteria (7, 8), attending the Spondyloarthritis Clinic of our Rheumatology Unit, in the period 1st June-7th August 2020, were recruited during regular outpatient clinic. Inclusion criteria were: age≥18 years old, ongoing therapy with biotechnological agents or small molecules. Exclusion criteria were: inability to sign informed consent, therapy with conventional synthetic DMARDs (csDMARDs) alone. Information on COVID-19-like symptoms since last visit, SpA disease activity, inflammation indices (C-reactive Protein-CRP, Erythrocyte Sedimentation Rate-ESR) was collected during the visit. Clinical examination was performed and blood samples for serology were collected. Disease activity was assessed by Ankylosing Spondylitis Disease Activity Score (ASDAS), 66/68 swollen/tender joint count, Disease Activity Score (DAS)-28. Patients with a positive serological test (either IgM or IgG or both) additionally underwent nasopharyngeal swab, analysed by RRT-PCR to detect SARS-CoV-2 genome and confirm active infection.

For comparison, the following sera were considered: 1) consecutive sera from healthcare workers (HCW) attending SpA patients or laboratory services at Padova University Hospital, and undergoing serology as screening, 2) consecutive sera from pre-COVID-19 era (2015).

### Serology

Serologic test for SARS-CoV-2 was performed by the MAGLUMI™ 2000 Plus (New Industries Biomedical Engineering Co., Ltd [Snibe], Shenzhen, China). This is a chemiluminescent analytical system (CLIA), featuring high throughput (up to 180 tests/h). According to the manufacturer’s inserts, the SARS-CoV-2 IgM cut-off is 1.0 AU/mL, while the SARS-CoV-2 IgG cut-off is 1.1 AU/mL. Manufacturers claimed that the calculated clinical sensitivities of IgM and IgG were 78.65% and 91.21%, respectively, while specificities of IgM and IgG were 97.50% and 97.3%, respectively. This assay was extensively validated in sera of COVID-19 patients at University Hospital of Padova (9).

The study complies with the Declaration of Helsinki. The Ethics Committee of the “Azienda Ospedaliera- Università degli Studi di Padova” (Padova, Italy) approved the research protocol and informed consent was obtained from the involved subjects.

### Statistical Analysis

Data distribution (normal or not normal) was verified through graphical representation (histograms, P-P plot, Q-Q plot) and then verified through Shapiro-Wilk normality test. Data were then represented as mean (standard deviation) in case of normal distribution, and median (interquartile range –IQR) in case of non-normal distribution, for continuous variables. Categorical variables were instead expressed as number (percentage). Baseline characteristics across the 3 groups were compared through Kruskal-Wallis (KW) test for independent samples in case of continuous variables and Chi-square (χ^2^) for categorical variables. Comparison between 2 groups were conducted by Wilcoxon rank sum/signed rank tests (as most data were not normally distributed) or for continuous variables, and by χ^2^ test for categorical variables, as appropriate. P values ≤ 0.05 were considered as significant.

## Results

A total of 396 patients underwent serological test for SARS-COV-2: 200 SpA patients, 95 HCW and 101 healthy controls. Baseline characteristics, including IgM/IgG levels, are represented in **Table 1**. All SpA patients were undergoing biotechnological therapies with anti-TNFα/IL-17/IL-23 drugs, or small molecules (mainly apremilast). About 28% were taking concomitant csDMARDs; only 5 patients were receiving steroids, all ≤ 5mg of prednisone equivalent/day. The mean ASDAS and DAS28 scores indicated a generally low disease activity.

**Table 1:** Baseline characteristics of the included populations.

SpA patients n=200	Characteristics
Age, mean (SD)	49.6 (14.7)
Male sex, n (%)	109 (54)
ASDAS, median (IQR)	1.76 (1.23-2.58)
DAS28, median (IQR)	2.33 (1.95-3.61)
CRP (mg/L), median (IQR)	2.9 (1.3-4)
ESR (mm/h), median (IQR)	8.0 (4.0-18.0)
Biotechnological treatment, n (%)	200 (100)
Anti-TNFα, n (%)	156 (78)
Anti-IL17, n (%)	19 (9)
Anti-IL12/23, n (%)	14 (7)
Small molecules, n (%)	11 (6)
Concomitant csDMARDs, n (%)	56 (28)
IgM anti SARS-CoV-2 KU/L, median (IQR)	0.21 (0.12-0.41)
IgG anti SARS-CoV-2 KU/L, median (IQR)	0.03 (0.02-0.05)
**Health Care Workers n=95**	
Age, mean (SD)	46.7 (12.9)
Male sex, n (%)	33 (35)
IgM anti SARS-CoV-2 KU/L, median (IQR)	0.38 (0.28, 0.43)
IgG anti SARS-CoV-2 KU/L, median (IQR)	0.03 (0.02, 0.06)
**Healthy Controls before 2015 n=101**	
Age, mean (SD)	50.6 (10.6)
Male sex, n (%)	63 (62)
IgM anti SARS-CoV-2 KU/L, median (IQR)	0.51 (0.45, 0.55)
IgG anti SARS-CoV-2 KU/L, median (IQR)	0.04 (0.02, 0.05)

Continuous data are presented as mean (SD) if normally distributed or median (IQR) if non-normally distributed; categorical data are presented as number of patients (%); ASDAS, Ankylosing Spondylitis Disease Activity Score; DAS28, Disease Activity Score; CRP, C-Reactive Protein; ESR, Erythrocyte Sedimentation Rate.

Anti-SARS-CoV-2 IgM levels were different across the 3 groups (KW χ^2^_2_ = 93.53, p=0.0001), with SpA patients having lower baseline levels; however, all median baseline value were well below the positivity range. IgG level were not significantly different across the 3 groups (KW χ^2^_2_ = 4.26, p=0.11).

In each group, the following number of patients presented with positive serology (either IgM or IgG, or both): 25 (12.5%) in SpA, 8 (8.4%) in HCW, 0 (0%) in healthy controls (χ^2 ^= 13.72, p=0.001). According to the post-hoc two-by-two comparison this difference was due to a statistically significant difference between SpA and healthy controls (χ^2 ^= 13.76, p<0.0001), and between HCW and healthy controls (χ^2 ^= 8.86, p=0.003), rather than a difference between SpA patients and HCW (χ^2^ = 1.08, p=0.30).

Among patients with a positive serology in SpA and HCW, there was a statistically significant difference in levels of IgM, which were higher in the SpA than in HCW (median (IQR) 1.53(1.19-3.08) versus 0.41(0.31-1.53) KU/L; Wilcoxon z = 2.39; p=0.016). On the contrary, IgG mean titres were lower in SpA (median (IQR): 0.04 (0.03-0.11) KU/L versus 1.22 (0.07-1.93), Wilcoxon z = -2.10, p=0.035). Among SpA patients with positive serology, 9 had a high IgM titre (>2.00 KU/L), and 2 a high IgG titre (>2.00 KU/L). In the HCW group, none of the patients had an IgM or IgG titre >2.00 KU/L.

Out of the 25 SpA patients with positive serology, only 5 (20%) reported COVID-like symptoms in the previous weeks. Interestingly, also 7 patients (4%) out of the 168 with negative serology reported COVID-like symptoms, which was significantly lower than those with positive serology (χ^2^ = 9.38, p=0.009).

Only two of the SpA patients with positive serology –both also reporting recent symptoms (within the past month)-, had a positive RT-PCR at nasopharyngeal swab, thus, following our evaluation, they underwent quarantine and subsequent re-testing until negativity. Of the 2 positive symptomatic patients, one had positive IgM (3.41 KU/L) with negative IgG (0.68 KU/L), while the other had both IgM and IgG positive (5.55 and 15.94 KU/L respectively).

In the HCW group, none of the patients with positive serology reported symptoms, and all of them had a negative RT-PCR.

**Figure 1:**
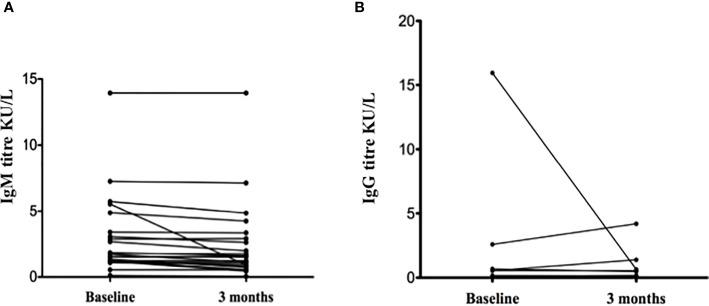
Anti-SARS-CoV-2 IgM **(A)** and IgG **(B)** levels in spondyloarthritis patients with positive serology (n = 25) (please note some lines might overlap).

In the SpA population with positive serology, at the 3 months control, the IgM level of each patients did not seem to change much in terms of absolute value, except in one patient (**Figure 1A** **)**. However, the mean IgM titre decreased from 2.76 ± 2.93 to 2.38 ± 2.95 (median 1.53 (1.19-3.08) to 1.22 (0.81- 2.93); Wilcoxon z=-3,47 p=0.001). As for IgG titres, they also remained overall stable. In only one patient (the one with overt infection and high IgG titre), the IgG levels dramatically decreased after 3 months (**Figure 1B**). The general trend of mean IgG titres decreased from 0.89 ± 3.25 to 0.31 ± 0.87 (median from 0.04 (0.03, 0.11) to 0.04 (0.03, 0.13); Wilcoxon z=0.17, p=0.86). No correlation was found between disease activity indexes and IgG or IgM levels at baseline or during follow up, in the whole SpA population or in seropositive patients only (data not shown).

## Discussion

The results of our pilot study showed that SpA patients undergoing cytokine-targeted treatment and HCW were exposed to SARS-CoV-2 and developed varying degrees of immune response against the virus. Overt COVID-19 infection occurred in 2/200 SpA patients. Given the absence of positive sera in the 2015 group, it seems unlikely that these findings can be related to false positive results. In addition, COVID-19-like symptoms were more frequently observed in those with a positive serology compared to the negative patients. None of the controls were symptomatic, or showed elevated anti-SARS-CoV-2 IgG/IgM antibodies. At 3 months, the mean IgM and IgG titres decreased in SpA patients.

Our findings highlight that serology is useful to detect previous contact with COVID-19 and could help estimate the extent of asymptomatic spread of the virus (10). Of course, the performances of SARS-CoV-2 serological tests also depend on sensitivity and specificity. The CLIA system used in this study appears to have an optimal specificity, albeit with a slightly lower sensitivity versus other types of serology tests such as enzyme-linked immunosorbent assay (ELISA) (11). This would suggest a low rate of false positive results in our samples, which seems to be corroborated by the absence of positive serology in the 2015 group. However, disease prevalence is another very important element to consider in a test’s performance, as it influences positive predictive value (PPV): a low disease prevalence (in this case COVID-19) decreases PPV (i.e., the chance that a positive result indicates true disease). Indeed, our serological testing has been conducted in a phase of relatively low COVID-19 prevalence: in the Veneto region, in the period in which the study was performed, the mean prevalence of COVID-19 was quite low (about 238 per 100.000 inhabitants) (12). Nonetheless, given that specificity, both reported by the manufacturer and by comparative studies, approached 100%, the estimated PPV—mainly influenced by specificity—still remains quite high (9). We may therefore assume that our positive results indeed indicate a contact with SARS-CoV-2, suggesting a much higher diffusion rate than previously reported in the timespan of interest. This would also be supported by the significantly more frequent COVID-19-like symptoms in SpA patients with a positive *vs.* negative serology. Such considerations may not apply in other rheumatic conditions such as systemic lupus erythematosus, where a positive serology might also stem from cross-reactivity with auto-antibodies (13). However, the pathogenetic mechanism of SpA is quite different, as it mainly involves innate immunity and autoantibodies are not an issue (14).

Importantly, no significant differences in the number of seropositive patients were found between SpA patients and HCW. Whether this means that SpA patients have similar seropositivity levels than general population is difficult to establish, especially since HCW are not a typical “general” population, due to a (theoretically) higher work-related exposure risk. However, literature indicates that patients affected rheumatic diseases seem to have the same rates of COVID-19 infection (diagnosed with nasopharyngeal swabs RT-PCR) (15). If this similarity will be confirmed in future serology studies as well, it will mean that immunosuppressed SpA patients are really as exposed as general population.

Regarding the potential role of cytokine-targeted therapies in SARS-CoV-2 susceptibility, it is very interesting to notice how none of the symptomatic patients had severe symptoms (respiratory insufficiency, fever>39°, organ failure). Moreover, in the 2 cases of documented infection in SpA, COVID-19 had a mild course and hospitalization was not required, in line with the literature describing mild/moderate disease course in immunosuppressed COVID-19 patients with arthritis (2, 16, 17). However, some authors pointed out that the infectious disease course might be also influenced by other factors, such as age, sex, comorbidities, and even the type of immunosuppressive treatment (11, 16). In fact, drugs targeting TNFα or IL-1 and 6 might have a beneficial effect, as these cytokines are involved in COVID-19 pathogenesis (18). In contrast, the role of anti-IL-17 drugs is controversial: while an autoptic study of COVID-19 patients suggested a pathogenic role for Th17 lymphocyte, thus a potential benefit of blocking Th17, other studies highlighted a more severe clinical course in patients treated with secukinumab (17, 19). Our SpA patients with documented COVID-19 were both on anti-TNFα therapy, and indeed -as mentioned- their disease course was mild, with symptoms like fever, malaise, myalgia, lasting only about a week.

A further important point is the type and duration of the humoral response that SARS-CoV-2 can elicit: it is still unclear how frequently neutralizing antibodies are produced in response to SARS-CoV-2 infection, and whether their decline is correlated with COVID-19 severity. In fact, while some authors underlined that in milder disease course there can be a faster antibody clearance, other studies showed persistently high levels of IgG in a broad range of COVID-19 cases, including the less severe (20, 21). In our SpA patients, we observed a decrease in IgM titres at 3 months, which was not accompanied by and an increase in IgG. On the contrary, in some patients IgG levels even decreased. This result seems to indicate a failure to develop an effective and prolonged immune response. Whether this result is dependent on a weak stimulation of the immune response due to low viral load, or on the interaction virus-host response, or on the use of anti-cytokine therapy remains to be elucidated.

The main limitation of our study is the small number of included patients: our intent was, however, to first perform a pilot study on the usefulness of serology, to then eventually apply it on a larger scale. Our plan would be to include a higher number of SpA patients, HCW, and adding a sample from general population, in order to able to take into account the role of important recognized confounders such as age, comorbidities, work-exposure and type of treatment. Cytokine and ferritin levels measurement could additionally add insight about the activation of the inflammatory cascade in SpA versus healthy individuals. The strengths are the inclusion of consecutive patients and the availability of controls, both in another at-risk population (HCW) and in sera collected before COVID-19 era.

In conclusion, serology revealed that exposure to COVID-19 in SpA patients, as well as HCW, was higher than expected based on reported symptoms or nasopharyngeal swab results. Targeted anti-cytokine therapy could act as a protective factor for a severe disease course, although this needs to be confirmed in larger studies. The effectiveness and duration of immune response also seems another important point to clarify, given that in our population we could not observe proper seroconversion with raised IgG titres. Further studies are warranted to better define the role of immunosuppressive therapy and other factors in the development of an effective and durable immune response.

## Data Availability Statement

The raw data supporting the conclusions of this article will be made available by the authors, without undue reservation.

## Ethics Statement

The studies involving human participants were reviewed and approved by Ethics Committee of the “Azienda Ospedaliera- Università degli Studi di Padova”, Padova, Italy. CESC code: 4930/AO/2. URC: AOP2073. The patients/participants provided their written informed consent to participate in this study.

## Author Contributions

AO contributed to study conception and design, acquisition, analysis and interpretation of data, and drafted the article. ML and CC participated in study conception and data acquisition. GC and AP contributed to data acquisition. AD contributed to analysis and interpretation of data. MP and RR designed the study and contributed to interpretation of data and data acquisition. All authors contributed to the article and approved the submitted version.

## Funding

This work was in part supported by a grant from Fondazione Cariparo (grant number 55813).

## Conflict of Interest

The authors declare that the research was conducted in the absence of any commercial or financial relationships that could be construed as a potential conflict of interest.
